# TBVAC2020: Advancing Tuberculosis Vaccines from Discovery to Clinical Development

**DOI:** 10.3389/fimmu.2017.01203

**Published:** 2017-10-04

**Authors:** Stefan H. E. Kaufmann, Hazel M. Dockrell, Nick Drager, Mei Mei Ho, Helen McShane, Olivier Neyrolles, Tom H. M. Ottenhoff, Brij Patel, Danielle Roordink, François Spertini, Steffen Stenger, Jelle Thole, Frank A. W. Verreck, Ann Williams, TBVAC2020 Consortium, Warwick Britton

**Affiliations:** ^1^Department of Immunology, Max Planck Institute for Infection Biology, Berlin, Germany; ^2^Immunology and Infection Department, London School of Hygiene and Tropical Medicine, London, United Kingdom; ^3^Tuberculosis Vaccine Initiative (TBVI), Lelystad, Netherlands; ^4^Bacteriology Division, MHRA-NIBSC, Potters Bar, United Kingdom; ^5^University of Oxford, Oxford, United Kingdom; ^6^Institut de Pharmacologie et de Biologie Structurale, IPBS, Université de Toulouse, CNRS, UPS, Toulouse, France; ^7^Leiden University Medical Center, Leiden, Netherlands; ^8^RegExcel Consulting Ltd, Surrey, United Kingdom; ^9^Centre Hospital Universitaire Vaudois, Lausanne, Switzerland; ^10^University Hospital of Ulm, Ulm, Germany; ^11^Biomedical Primate Research Centre, Rijswijk, Netherlands; ^12^Public Health England, London, United Kingdom

**Keywords:** tuberculosis, bacille Calmette–Guérin, vaccination, biomarker, clinical trial, portfolio management, discovery

## Abstract

TBVAC2020 is a research project supported by the Horizon 2020 program of the European Commission (EC). It aims at the discovery and development of novel tuberculosis (TB) vaccines from preclinical research projects to early clinical assessment. The project builds on previous collaborations from 1998 onwards funded through the EC framework programs FP5, FP6, and FP7. It has succeeded in attracting new partners from outstanding laboratories from all over the world, now totaling 40 institutions. Next to the development of novel vaccines, TB biomarker development is also considered an important asset to facilitate rational vaccine selection and development. In addition, TBVAC2020 offers portfolio management that provides selection criteria for entry, gating, and priority settings of novel vaccines at an early developmental stage. The TBVAC2020 consortium coordinated by TBVI facilitates collaboration and early data sharing between partners with the common aim of working toward the development of an effective TB vaccine. Close links with funders and other consortia with shared interests further contribute to this goal.

## Introduction

One hundred years ago, Albert Calmette (1863–1933) and Camille Guérin (1872–1961) had almost reached their goal (1). They had passaged *Mycobacterium bovis* 200 times at 14 d intervals on potato slices soaked with ox gall in their attempt to generate an attenuated vaccine against tuberculosis (TB). They had started their program in 1906 and had already obtained preliminary evidence for safety and protection of their candidate in 1913. Yet, they continued until they had completed the 230th passage before verifying its efficacy and safety in experimental animals including guinea pigs, rabbits and non-human primates (NHPs) as well as in the natural host, cattle. The data obtained were highly promising so Calmette and Guérin started the first vaccination of a human neonate born into a household with a TB patient. Risk of TB in a household-contact infant at those times was extremely high. Yet, the baby did not develop TB and after additional encouraging results, vaccinations of more than 20,000 neonates in households with at least one TB patient were performed between 1921 and 1924. The results of this trial revealed that of the vaccinees 5% had died and 1% of these of TB, whereas knowledge of the time held that a quarter of non-vaccinated newborns would die in the first years of life, many of them of TB (1). This is the start of Bacille Bilié Calmette-Guérin, later shortened to Bacille Calmette–Guérin (BCG), which remains the only licensed vaccine against TB until today. Although not perfect, this vaccine partially fulfills the goal set by Calmette and Guérin, since it protects against extra-pulmonary forms of disease in infants, including life threatening TB meningitis. Unfortunately, in most settings, BCG does not reliably prevent pulmonary TB, the most prevalent and the most contagious form in all age groups from the neonatal to the elderly population. The lack of an efficient vaccine against pulmonary TB is a major obstacle to control TB satisfactorily until today.

In 1993, the World Health Organization (WHO) declared TB a global emergency at a time when some 4–6 million new TB cases per year were notified. With the introduction of a new treatment regimen, called directly observed treatment, short-course (DOTS) program, WHO claimed to bring TB under control and predicted a decline from 6–8 million new cases in 1997 to 2–3 million new cases by 2017 by implementing highly intensive DOTS (2). Unfortunately, this hope failed miserably. The most recent notifications reveal a stunning 10.4 million new cases and 1.8 million deaths in 2015. Building on better drug regimens alone not only proved to be insufficient in controlling TB, it also led to increasing incidences of multi-drug-resistant forms of TB.

At this time, researchers interested in the mechanisms underlying immunopathology and immune protection of TB were concerned that the efficacy of DOTs might be overestimated and considered a better TB vaccine a necessity for efficient TB control. Thus, a proposal on TB vaccines was submitted to the European Commission (EC) funded framework program (FP) 5 and this was funded from 1998 to 2002. The subsequent FP 6 and FP 7 programs continued to support TB vaccine-related research until 2013. During this period, enormous progress was achieved, not only resulting in the development of a number of promising vaccine candidates but also providing deeper understanding of immune mechanisms underlying protection and pathology in TB as well as identifying new TB biomarkers (BMs). These FP5 to FP7 activities were succeeded by the Horizon 2020 program which further supported TB vaccine research and development. It is this continuous support over the past 20 years which has welded together researchers from all over Europe and other parts of the globe to harness cutting-edge knowledge in the immunology, microbiology, and high end technology platforms for the development of novel TB vaccine candidates. This sustained EC funding made it possible to establish a unique European consortium responsible for most of the global innovation in TB research.

This EC-funded activity proved extremely successful. Over 50% of the global pipeline in TB vaccine candidates currently evaluated in clinical trials originate from EC funded projects (3). The EC has played a key leadership role in accelerating TB vaccine research and development and ensured a well-coordinated and efficient TB vaccine research consortium.

At the same time, the pipeline needs to be fed constantly with new innovative candidates since it is unlikely that one single vaccine could protect against all different forms of TB disease in all age groups and for all indications. Thus, the concept of diversification gained increasing importance. Different vaccine types (subunit vaccines vs. whole cell vaccines), different administration schedules (pre-exposure vs. post-exposure), different age groups (neonates vs. adults) and different purposes (prevention of infection vs. disease vs. recurrence of disease), and perhaps even therapeutic TB vaccines have all to be considered.

**Figure 1:**
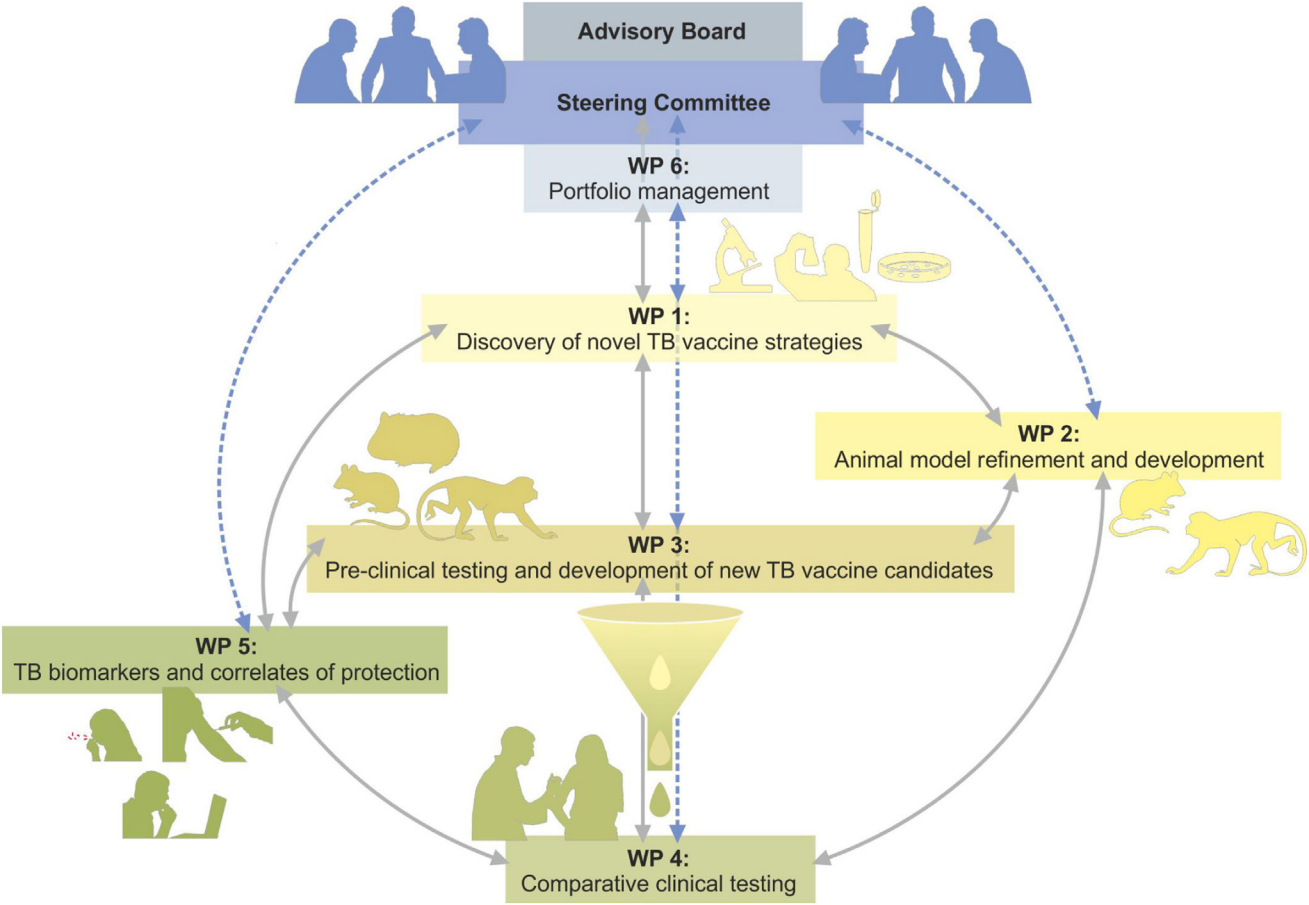
Overview of the TBVAC2020 project structure indication interactions between WorkPackages (WPs) and flow from discovery *via* preclinical testing to clinical trial of most promising vaccine candidates.

Already before the beginning of TBVAC2020, it was realized that an isolated vaccine development approach was inadequate and needed to be complemented by deeper knowledge of patho-mechanisms, immunology, and microbiology; by established as well as novel animal models; by identifying biosignatures of TB disease, risk of disease, vaccine efficacy, and safety (3–6). Finally, with increasing numbers of vaccines being ready for clinical testing, early clinical trials needed to be integrated. Although TBVAC2020 and its predecessors respect individual researchers’ autonomy, it was deemed of increasing importance to also provide a portfolio management process, including consensus gating criteria, to objectively and transparently guide further product and clinical development of TB vaccine candidates (7). This article describes the different activities of the Horizon 2020 funded TBVAC2020 project which is coordinated by TBVI summarizing recent achievements and future goals as part of the European effort to successfully combat TB. Figure 1 provides an overview of the close interactions between different WorkPackage (WP) activities and progression of promising vaccine candidates from discovery *via* preclinical testing to clinical trial. Although TBVI has coordinated much of the European research efforts on TB vaccine development to date, there are other ongoing activities, for example, through the Aeras Foundation^1^ and the EMI-TB Consortium.^2^

## WP1 Discovery of Novel TB Vaccine Strategies

A major objective of TBVAC2020 is the identification of novel vaccine candidates for subsequent preclinical and early clinical development. To this end, the objective of WP1 is to develop innovative approaches and platforms for TB vaccination in three areas: (i) the discovery of novel antigens and live vaccines; (ii) the exploration and implementation of novel immunization strategies and vaccination platforms; and (iii) the optimization of formulation and delivery of available subunit and live vaccine candidates.

### Antigen Discovery

The identification of novel antigens is the initial and critical step in developing new vaccination strategies. It is well established that polyfunctional effector T cell subsets act in concert to activate macrophages to limit mycobacterial replication and prevent progression of latent infection to active disease. Our key objective is, therefore, to identify antigens that trigger protective lymphocyte subsets and to improve their delivery to antigen-presenting cells. Specifically, this sub-work package aims to identify: (i) new immunogenic epitopes derived from *Mycobacterium tuberculosis* (*Mtb)*-infected macrophages; (ii) new immunogenic proteins and lipoproteins of *Mtb*; (iii) new immunogenic lipids of *Mtb*; and (iv) antigens that induce antibody-mediated protection.

A high level of innovation is being achieved by applying sophisticated techniques, e.g., for the elution of peptides from human leukocyte antigen (HLA)-molecules from *Mtb*-infected antigen-presenting cells using a data-independent acquisition (DIA) method which aims to complement traditional mass spectrometry-based proteomics techniques and SRM methods (SWATH-MS) (8). This unbiased approach allows the identification of immunogenic antigens that are presented during natural infection with *Mtb*. The use of transcriptomic analysis is another unbiased approach to identify antigens expressed at the site of infection. Unbiased transcriptomic approaches were also used to identify antigens expressed at the site of infection. A number of new *in vivo* expressed (IVE) *Mtb* antigens were identified that were highly expressed in the lungs of TB susceptible (C3HeB/FeJ) mice. Using various predictive algorithms, including potential HLA-I and -II presented epitopes many new antigens were identified that activated both conventional and unconventional T-cells from latently infected individuals (9). Of note, many IVE-TB antigen-directed T cell responses were characterized by the production of cytokines other than IFNγ. Studies are ongoing to dissect antibody recognition of this novel class of antigens.

In parallel to these unbiased approaches, WP1 is following hypothesis-driven approaches for the discovery of vaccine candidates. For example, stage-specific, subdominant mycobacterial antigens encoded by genes co-regulated with those encoding latency antigens, such as the heparin-binding hemagglutinin (HBHA) (10) are being evaluated. Recently, the importance of *Mtb*-specific chemokine receptor expression (CXCR3^+^ and CXCR5^+^) lung-homing T lymphocytes in protection against TB has been demonstrated (11). This knowledge has been exploited to show that the liposome-adjuvanted fusion protein H56/CAF01 confers durable protection against TB by eliciting protective T-cells expressing CXCR3 in the lung, while limiting the induction of non-protective intravascular T-cells (12). The identification and evaluation of lipid antigens is another key objective of WP1. Using protease cleavable CD1b-molecules, formulation of lipid-containing liposomes (13) or inclusion of α-galactosyl ceramide (α-GalCer) (14) to activate iNKT cells the potential of lipid antigens as vaccines is being evaluated. Liposomes are a particularly attractive delivery system, because they offer a simple and flexible platform to combine antigens and adjuvants, including immune modulators triggering the toll-like receptor (TLR) pathways (15).

Another innovative concept addressed in WP1 is the hitherto almost completely neglected search for *Mtb* antigens that trigger protection-associated antibody responses (16). To address this issue, *Mtb*-specific antibodies are being isolated from patients and immunized mice, and their mycobacterial targets are being identified.

### Delivery Systems and Immunization Strategies

#### Mucosal Delivery

The induction of protective immune responses in the lung, which is the site of *Mtb*-entry, is elementary for vaccine development. Previous data showed that BCG vaccination *via* the pulmonary route confers superior protection when compared to the subcutaneous route, which relies at least in part on Th17 cells (17–19). Mimicking the natural route of infection, the protective efficacy of whole-cell live-attenuated *Mtb* Δ*phoP*Δ*fadD26* (MTBVAC) delivered intranasally is being explored. A specific lung-targeted approach is to develop an inert pulmonary delivery platform, which can “pull” antigen-specific Th17 cells from the circulation into the lungs to form a depot of tissue-resident memory cells.

#### Designing Novel Adjuvants

Local pulmonary immunity could also be induced by parenteral delivery of adjuvants designed to promote site-specific homing of protective T cells. In this regard CD4^+^CXCR5^+^ T-cells promote early and efficient macrophage activation, and their recruitment requires a pre-existing Th17 response (20). This knowledge is translated into a new strategy by implementation of the Th17-inducing adjuvant, CAF01, combined with stimulators of CXCR5, CXCR3, and CCR4 known to favor pulmonary homing.

Synthetic glycolipids also have potent adjuvant activity and homologs of various mycobacterial glycolipids induce the inflammasome and have adjuvant activity *in vivo* (21). In particular, vaccination of mice with recombinant PPE44 formulated in dimethyldioctadecylammonium and synthetic glucose monomycolate (GMM) induced protective immunity against *Mtb*, with comparable efficacy to BCG. Induction of CD1b-restricted GMM-specific responses by this formulation is currently being tested in guinea pigs.

#### Developing Novel Inert and Viral Vector-Based Delivery Systems

Pre-existing anti-*Mtb* antibodies in the lung mucosa have also been suggested as a strategy to prevent infection, but so far it has been difficult to induce high-titer and long-lived antibody responses against structural antigens. A novel nanoparticle-based technology is being exploited for the delivery of synthetic cell wall antigens, a technology that has shown excellent results in the field of influenza (22). Improved viral vectors are currently being evaluated in WP1, including non-integrative lentiviral vectors (LV), chimpanzee adenoviral (ChAd) vectors, lymphocytic choriomeningitis virus and influenza virus. In particular, a vaccine regime using two viral vectors has been optimized: a ChAd vector (ChAdOx1) and a modified vaccinia virus Ankara (MVA), both expressing Ag85A. A boost of BCG with intranasal (i.n.) ChAdOx1.85A followed by a second boost with i.n. or intradermal MVA85A, significantly improved BCG vaccination in mice (23).

### Novel Live Vaccines

Past research programs (TBVAC and NEWTBVAC) have succeeded in developing novel live vaccines, including BCGΔ*ureC*:*hly* (VPM1002) (24), MTBVAC (25, 26), which already progress through clinical trials, and BCGΔ*zmp1* which is considered for clinical testing in the near future. One of the aims of WP1 is to build upon these first-generation live vaccines as well as to provide novel live vaccine candidates based on strong scientific rationale.

*Mtb* Δ*phoP*Δ*fadD26* will be used as a basis to search for safer and more effective mycobacteria-based vaccines to be used in immune-compromised individuals. Specifically, an additional virulence gene, *erp*, has been deleted from MTBVAC resulting in increased attenuation (27). To improve protection by MTBVAC, a genome-wide screening approach is being performed, in which random mutants are screened in mice for safety and protective efficacy. Deletion of the anti-apoptotic factor *nuoG* in VPM1002 resulted in improved safety and protection as compared to BCG and VPM1002 in the mouse model (28). A safer VPM1002 derivative was created by deletion of *pdx1* rendering the vaccine auxotrophic for vitamin B6 (29). Finally, an attenuated *Mtb* ESX-5 mutant, *MtbΔppe25-pe19*, is safe and more protective than BCG in mice (30). The mechanism of attenuation is yet unknown, but likely results from phagosomal-rupture-induced innate immune signaling.

To summarize, WP1 has identified promising novel adjuvants, delivery systems, and live vaccine candidates that are currently evaluated for safety, immunogenicity, and protective efficacy in appropriate animal models, in collaboration with partners in other WPs.

## WP2 Animal Model Refinement and Development

In the absence of unambiguous markers that correlate with and, thus, predict vaccine-induced protective immunity against *Mtb* infection and TB disease (31, 32), experimental infection models remain an indispensable tool in the process of TB vaccine research and development. And while at present no animal-free systems exist that fully recapitulate the complexity of host defense cascades and pathogenesis (33), it is imperative to exploit animals at various (staggered) stages of this vaccine Research and Development (R&D) process and in particular to demonstrate vaccine efficacy (34).

Typically, several lower and also higher vertebrate animal species are utilized as models from as early as discovery up to building qualification dossiers and down selecting the best candidate vaccines toward resource-consuming, advanced stage clinical testing (Phase II and later) (35). These models are not only used to investigate the protective efficacy against an infectious challenge but also to assess vaccine tolerability (or, in specific cases, attenuation of live vaccines as a parameter of vaccine safety) and immunogenicity of new approaches, which build upon or extend beyond the current BCG vaccine.

Strictly speaking, no single animal model has been validated for the ultimate predictive assessment of TB vaccines toward the various target populations that can be identified globally. Models by definition imply a simplification of real-life complexity. Especially in the light of developing new preventive/prophylactic vaccine strategies, we, therefore, explore candidate vaccine performance in several different models to strengthen its proof-of-concept and to de-risk the development process for possible failures (35). In recognition of the undoubted limitations of any specific model, centralized and independent preclinical evaluation modalities are established in mouse, guinea pig, and NHP hosts, as described in WP3.1.

Mostly, preclinical studies assess the prophylactic profile of vaccine candidates in healthy animal hosts, which are experimentally challenged with *Mtb* and that are immunologically naive to mycobacterial antigens and typically free from any other disease or infectious pressure. In the clinic, pre-exposure, co-infection, and/or co-morbidity conditions can be expected to have an impact on susceptibility and also vaccine efficacy (36). The TBVAC2020 consortium, which at this point just passed mid-term of its 4-year work program, has set out in a specific WP to develop and refine models toward various clinically relevant conditions. Grossly, we have defined three areas as follows: (A) modeling vaccination conditions post-*Mtb* exposure, (B) modeling disease risk factors and clinically relevant conditions, and (C) refinement of existing models. With an eventual goal of implementing alternative models to provide proof-of-concept in support of preclinical vaccine development and candidate portfolio management, animal studies are explicitly useful also for investigating fundamental aspects of TB infection and immunity and may contribute to the identification and validation of BM candidates as one of the other key objectives of this consortium (covered in WP5).

### Modeling Vaccination Conditions Post-*Mtb* Exposure

From the first year of the program onward activities have been initiated (i) to test alternative approaches to provoke reactivation of low-dose disease in mice and in the face of vaccination, (ii) to set up a so-called Cornell-type of post-exposure vaccination in guinea pigs, and (iii) to evaluate *Mtb* infection in cattle as a novel model of latent *Mtb* infection for post-exposure vaccine evaluation.

While both spontaneous reactivation and immunosuppressive regimens pose shortcomings in the context of modeling and testing efficacy of post-exposure vaccination strategies, we have explored “energy deprivation” by protein malnutrition (PEM) as an alternative to reactivate TB in mice. It was demonstrated that PEM, both in the infection and the vaccination phase, abrogates vaccine-induced protection, which is reversible by protein supplementation (37). Abrogation of vaccine efficacy correlated with the loss of cytokine positive memory populations from the periphery and in particular with the loss of IL2 producing CD4^+^ T cell subsets. Although prototype vaccination did overcome and protect from malnutrition-induced reactivation, the results also suggested that more stringent malnutrition conditions would be required to lend more robustness to this model. In the light of animal welfare, however, this line will not be pursued. In the guinea pig model, the test conditions for *in vivo* bacterial expansion (regrowth) after antibiotic treatment have been established. The dynamic window of this model has been established using RUTI™ as a therapeutic vaccine candidate (38), with promising results that merit a follow-up study testing other candidates. This model may also be a valuable tool for studying and developing TB treatment regimes toward drug-resistant TB. Using *Mtb* (rather than *M.bovis*) to infect cattle provides great potential for obtaining a large (outbred) animal model that is hall-marked by latent, low burden *Mtb* infection and that can be manipulated to induce disease reactivation (39). Although in its early days, if successful, this effort may provide a platform for analyzing vaccine efficacy in a state of latent TB infection and a perspective on identification of associated BMs.

### Toward Modeling of Risk Factors and Clinically Relevant Conditions

The workplan comprises the following conditions: (i) exploring differential monocyte/myeloid phenotypes in mice as a parameter of disease susceptibility, (ii) co-morbidity of obesity either or not in inflammation-prone mice and with multiple consecutive challenge conditions, (iii) stringent mouse modeling against clinical (Beijing-type) isolates of *Mtb*, and (iv) simian immunodeficiency virus co-infection in NHPs.

While monocyte frequency and phenotype have been associated with TB risk in the clinic (40–42), different strains of mice, which express differential profiles of myeloid cell compartment markers, have been used in BCG vaccination and protection experiments. In the face of variable efficacy in a selected range of host strains (43), flow cytometric analyses allow correlation of monocyte numbers and (functional) phenotypes with BCG-induced efficacy. Also in mice, we have exploited commercial high-fat diet to induce obesity (not formally characterized as a diabetic condition, yet showing impaired glucose tolerance). In a matrix-type study design, varying parameters relating to obesity, low-dose aerosol infection and multiple consecutive infections, disease susceptibility, and standard BCG efficacy are investigated. Further to experimental TB modeling in mice, multiple clinical isolates with different clinical and genotypic appearance were cultured and selected from Beijing, Beijing subtype and non-Beijing strains. Supported by molecular typing and *in vitro* virulence analyses using bone-marrow-derived macrophage cultures, seed lots were prepared for future vaccine evaluation testing from a limited number of strains, as well as a standard laboratory strain for comparison. Mouse infection experiments further corroborated the notion that HN878 and M2 strains provide highly pathogenic phenotypes both *in vitro* and *in vivo* (44, 45). A study toward the definition of a TB/HIV co-infection model for vaccine evaluation using TB/SIV co-infection in NHP is in progress. All cynomolgus macaques in the study controlled a stable low grade chronic *Mtb* infection for 16 weeks prior to SIV challenge. Co-infection with SIV leads to a reduction in CD4 T cells and trends for higher disease burden with mycobacteria-specific and SIV-specific immune responses detected in all co-infected macaques.

### Refinement of Existing Models

To strengthen the use of the well-established guinea pig model in TB vaccine R&D, methods have been developed to quantify and characterize antigen-specific T cell responses. Specific T lymphocytes are expanded, isolated, and re-stimulated to investigate functional markers by quantification of cytokine and other effector molecule expression levels. Further vaccine experiments will assess anti-mycobacterial immunity by these new tools in this stringent model. This refinement is particularly relevant for the analysis of unconventional vaccine candidates based on mycobacterial lipid antigens. Guinea pigs, in contrast to mice, do express a functional CD1 type 1 antigen presentation system, thus, representing the only small animal model to study these unconventional vaccine research approaches.

As another means of refinement, new NHP modeling tools and conditions are being established for refined infectious challenge, advanced flow cytometric analyses (including that of innate subsets), and advanced imaging of disease and host response dynamics by PET/CT imaging (46). To the latter in particular, the consortium also collaborates with external partners in a global network [governed by the Bill & Melinda Gates Foundation (BMGF)^3^]. The work in NHP so far has revealed pro- vs. anti-inflammatory/regulatory signatures that correlate with disease susceptibility and that hint toward a key role for differential innate immune orchestration. While, typically, NHP studies and protective efficacy assessment rely on measuring peripheral immunity and parameters of disease reduction, we currently apply standard and non-conventional vaccination to investigate early and local, innate and adaptive immune response parameters, and toward possible proof-of-concept for protection against infection (rather than protection against disease) in this highly susceptible species.

Overall, new modalities for modeling reactivation of disease (rather than primary infection) have been evaluated and are ongoing in different species (mouse, guinea pig, and cattle). More stringent and clinically relevant conditions for preclinical *in vivo* analysis of vaccines have been established. Benchmarking by standard/prototype vaccination is ongoing in mice, guinea pigs, and NHP toward diversification and refinement of preclinical vaccine R&D strategies. All these activities, as a two-pronged attack, add to our continuous and concerted effort to strengthen preclinical evaluations of candidates as well as our basic understanding of pathogen–host interaction in TB.

## WP3 Preclinical Testing and Development of New TB Vaccine Candidates

The overall aim of WP3 is to support and facilitate the progression of vaccine candidates from discovery through to preclinical development. This is achieved in two sub-WPs. WP3.1 enables the selection of successful candidates through comparative head-to-head testing in standardized animal models. These studies provide crucial information for vaccine developers and also support activities in WP3.2 which aim to take the prioritized, most promising preclinical TB vaccine candidates and accelerate their advancement through the TB vaccine pipeline. WP3.2 provides expertise in all aspects of product development which enables relevant advice *via* tailor-made Product and Clinical Development Teams (P&CDTs) to be given to developers in order to advance their candidates in an efficient manner. Specific issues are identified and guidance/recommendations are given to developers. Close interaction between the sub-WPs is essential to ensure effective use of resources and this is facilitated by the Portfolio Management Committee (PMC) (WP6).

## WP3.1 Head-to-Head Testing in Standardized Preclinical Models

Demonstration of the safety, immunogenicity, and efficacy of vaccine candidates in animal models is required for preclinical development. This WP aims to provide evidence that the candidate is safe and elicits:

- an immune response that can be justified as being anti-mycobacterial and is of appropriate magnitude in terms of strength and duration, compared to a benchmark such as BCG;
- a protective effect against experimental challenge with *Mtb* which is demonstrated by a quantifiable reduction in disease burden compared to no treatment and/or BCG or another relevant benchmark vaccine.

Systematic, independent, and objective comparison of TB vaccines in well-characterized animal models enables the identification of the vaccines that have the greatest potential to be immunogenic and to show efficacy in the clinic. WP3.1 provides capacity for animal testing, building upon the concept that was developed in previous FP5, FP6, and FP7 projects for head-to-head comparisons of candidates in standardized animal models. In addition, there is an aim to offer models that better represent the target product profiles of the vaccines being developed.

The objective of WP3.1 is to compare and prioritize vaccines from the candidate discovery work package of this project (WP1) which supports decision-making at the individual partner and WP level and provides objective evidence for decision-making in portfolio management using the stage-gating criteria (WP6). Wherever feasible, samples are retained for selected evaluation of potential correlates of protection (CoP; WP5). The partners providing the models all have experience and a track record in TB R&D and importantly are recognized providers of unbiased, high quality evaluation of vaccines in their respective models. A summary of each of the models provided is given below.

### SCID Mouse Model for Safety of Live Vaccines

Assessing the residual virulence of live vaccine candidates in the context of an immunodeficient host is an essential part of modern pre-clinical vaccine evaluation. Vaccine candidates are administered into severe combined immune deficient (SCID) mice using methodologies and study designs described (47). This has become an accepted standard for safety evaluation of live vaccines (48) and has been successfully used to benchmark the safety of live vaccine candidates against BCG.

### Standard Immune Competent Mouse Model for Protective Efficacy

The first screen of vaccine candidates for immunogenicity and efficacy is usually performed by individual laboratories during the discovery phase. Capacity has been provided in WP3.1 for testing in a standard mouse model to assist laboratories, which lack facilities for *Mtb* challenge studies, and/or to facilitate decision-making within the project. For example, within WP1 where different adjuvants and delivery systems are being developed, a head-to-head comparison could allow prioritization based on protective efficacy. The model entails vaccination of CB6F1 (H-2^b/d^) hybrid mice, subsequently challenged with *Mtb* H37Rv *via* the respiratory route with protection determined by bacterial load in lungs and spleen at 6 weeks post infection.

### Mouse Model for Protective Efficacy against Clinical (Beijing) Strains of *Mtb*

It is important to demonstrate that new TB vaccine candidates can protect against challenge with clinically relevant and highly virulent strains of *Mtb*, including Beijing strains, which are prevalent in East Asia. Highly virulent *Mtb* strains (one Beijing strain and one Euro-American lineage, see also activities in WP2), showing a lower efficacy of the BCG vaccine (49), are used to challenge by inhalation, vaccinated mice (either pre- or post-exposure). Protection is determined at 4 weeks and 10 weeks after challenge.

### Mouse Model for Efficacy of Vaccine Candidates Given Post-Exposure

Robust animal models are needed to be able to test that vaccines can be safe and efficacious in individuals who are already infected with *Mtb*. A well-established mouse model for therapeutic vaccination is provided. This model has been validated previously (50, 51). The model involves aerosol infection followed by chemotherapy administered for 8 to 10 weeks post-infection with vaccination within weeks 12–17 and efficacy determined at week 23.

### Guinea Pig Model for Protective Efficacy

The aerosol infection guinea pig model of TB has been widely used to determine vaccine efficacy, being regarded as a more stringent test than the standard mouse model. Data generated in previous EU-funded consortia demonstrated the utility of a standardized model, where the main read-out of efficacy is reduction in bacterial load, compared to control groups at 4 weeks post-challenge (47).

### Mouse Immunogenicity for Formulation Studies

In combination with a vaccine formulation service where TB vaccine candidates are optimized with various type of adjuvants, there is provision of immunogenicity data in a harmonized mouse model which enables rational selection through comparative assessment of promising adjuvanted TB vaccine candidates.

To provide further flexibility in the preclinical evaluation of candidates, an aim of the project is to develop new animal models and improve read-outs of existing models such that they recapitulate a broader range of relevant conditions as they appear in the human populations being targeted. This model development is being conducted in WP2 with a view to incorporate a newly developed animal model or readouts into WP3.1 in order to offer a wider range of head-to-head testing.

## Progress to Date

Up to June 2017, 51 tests (comprised of 21 different vaccine candidates/approaches) of safety, immunogenicity and/or efficacy have been completed (33 tests) or are on-going (18 tests) in the various models. Outcomes of completed studies are summarized below:

- In the standard mouse or guinea pig challenge models, three candidates have shown protective efficacy and four were not efficacious.
- In post-exposure or Beijing challenge models, eight candidates afforded significant protection, four gave partial protection and three did not protect.
- Safety data on four candidates using the SCID mouse model, showed that three were attenuated.
- Data on the stability and immunogenicity of seven adjuvant formulations have been provided for one antigen.

These data (both positive and negative) have informed the portfolio management (WP6) and supported the vaccine discovery activities (WP1).

## WP3.2 Preclinical Development

The main objective of this sub work package is to manage and facilitate the advance of the preclinical development pathway of new TB vaccine candidates. It supports the progression of the pipeline by advancing candidates prioritized through preclinical toward an early clinical stage of development using a portfolio management approach (as described in WP6). Tailor-made P&CDTs, including experts in regulatory, manufacturing, and clinical studies of vaccines, are established together with the selected vaccine developers. Currently, there are four vaccine candidates in preclinical development. For HBHA and BCGΔ*zmp1* candidates, joint P&CDTs with Aeras (as part of the joint global effort in TB vaccine development) have also been established to support these candidates in the context of the activities of this project. More information about the P&CDT can be found in the chapter describing WP6.

### Heparin-Binding Hemagglutinin

Heparin-binding hemagglutinin (52, 53) in combination with adjuvant giving the best level of immunogenicity and protection in animal models and in *ex vivo* models are compared and evaluated, in order to select the best combination. For vaccine optimization, a thermo-stabilized form of HBHA, which will make it less dependent on the cold chain and, therefore, more logistically accessible for most of the TB endemic countries, will also be developed and evaluated.

### Recombinant BCGΔzmp1

Confirmation of identity, drug susceptibility and genetic stability of BCGΔ*zmp1* mutant (54, 55) strain(s) over serial passages (>12) has been achieved. An unmarked Δ*zmp1* mutant in a background strain with full background history and freedom to operate in terms of product development to licensure has been generated and characterized with respect to safety, immunogenicity and efficacy in various animal models within the consortium.

### Combination of M72/AS01_E_ and ChAd3M72

The M72 vaccine, containing both human CD4^+^ and CD8^+^ T cell epitopes, is an adjuvanted (AS01E) fusion protein vaccine candidate and has been evaluated in clinical trials (56, 57). The use of ChAd vector has been shown to elicit strong CD8^+^ T cell responses. The ChAd3M72, using a viral vector for delivery, is developed as a boost for M72/AS01E prime. This heterologous prime boost approach has been assessed in a mouse model to induce a higher number of antigen-specific CD4^+^ and CD8^+^ T cells in the lung and peripheral blood with a different profile of CD4^+^ T cell polyfunctionality as compared to two doses of M72/AS01E (unpublished data).

### MVA–TB Vaccine Candidates

Preclinical development of the MVA vectors expressing multiple *Mtb* antigens specifically targeted as a therapeutic vaccine is to improve treatment of active TB (in particular linked to drug-resistant strains) and to prevent reactivation and/or re-infection in the adult population, in particular from endemic countries. Genetically stable MVA–TB vaccine candidates (58) are currently being evaluated using different murine post-exposure models (with collaborators in the USA and Spain). In addition, a manufacturing process for MVA–TB from a cell line and not primary cell cultures (NIH grant with Emergent BioSolutions) is also under development.

*Additional support service is provided* for specific vaccine formulation optimization and characterization of TB vaccine candidates selected within the consortium (59). A panel of adjuvants (including emulsions, liposomes, aluminum salts, TLR agonists, and others) and different production methods are available for formulation. This will allow vaccine developers to investigate and improve the compatibility and stability of their antigen with different adjuvants, and to define the optimal production methods to generate physico-chemically stable and immunogenic TB vaccine candidates.

WorkPackage 3 provides a valuable resource for vaccine developers especially those from an academic environment to progress their vaccine candidates from discovery to the preclinical development stage. The developers with selected vaccine candidates have access to various preclinical animal models and the support of the P&CDT. The advice from the tailor-made P&CDT is not restricted to regulatory, manufacturing, and early clinical studies, but also includes project management. These activities will lead to a more diverse and more promising pipeline of novel vaccine candidates at preclinical stages, and subsequently also for early clinical stages.

## WP4 Comparative Clinical Testing in TBVAC2020

Early phase I and first-in-man clinical testing is an essential bridge between the demonstration of safety and efficacy in preclinical animal models and field testing in clinical trials in TB endemic countries. Such early clinical testing focuses on evaluating safety, usually in healthy adults, and immunogenicity, using a variety of standardized immunological assays. The overall concept underpinning WP4 is that standardized early clinical testing is essential, given the increasing numbers of diverse candidate TB vaccines being evaluated in preclinical animal models. There is currently a significant portfolio of diverse candidate vaccines in clinical and late preclinical development, which illustrates the progress made over the last 15 years (60). With the increasing number of diverse candidate vaccines currently in preclinical development, it is important to develop standardized methods and facilities for early clinical testing, in order that the most promising candidates can be selected for progression. There will never be sufficient resources to progress all the current candidates through to field testing in TB endemic countries. The use of standardized clinical trial protocols, standardized safety reporting, and standardized immunological evaluation according to published recommendations will allow comparison between different candidate vaccines (61, 62). This comparison will facilitate the rational selection of which candidates should be progressed to further clinical testing and efficacy testing in field trials. The aim of WP4 is to focus on early clinical TB vaccine development and to build on the successful standardized preclinical vaccine testing developed in EC supported FP6 TBVAC and FP7 NEWTBVAC projects. Over the last 10 years, the TBVAC/NEWTBVAC consortia have conducted many standardized head-to-head preclinical animal studies in mice, guinea pigs and NHPs. The data arising from these studies have facilitated vaccine selection (63, 64). This WP takes this successful approach a step further and applies it to early clinical testing.

Furthermore, there is an opportunity provided by this WP to embrace human experimental medicine studies, where early clinical trials can be used to demonstrate proof-of-concept. An example of this would be the evaluation of the safety and immunogenicity of a recombinant viral vector delivered by aerosol, compared with systemic administration (65).

## Process for Vaccine Selection

Vaccine candidates are selected for inclusion in this WP by the PMC, in conjunction with the TBVI P&CDT. This advice is independent of the vaccine developer. TBVI and Aeras have agreed on a set of portfolio management criteria which include stage-gating criteria (7, 34), and these criteria are used to guide vaccine selection in this WP4. The data arising from the trials conducted as part of this WP4 will be reviewed by the PMC for subsequent decisions about progression to safety and immunogenicity trials in TB high burden countries. Ongoing interactions with The European & Developing Countries Clinical Trials Partnership (EDCTP) and clinical sites in Africa, including those that are part of the EDCTP Networks of Excellence, will ensure that, subject to full and independent peer review, promising candidates are progressed as efficiently and effectively as possible in an endemic setting. Support and advice by tailor-made P&CDT will be provided to each vaccine developer to accelerate the development of the respective vaccine candidates through phase I/first-in-man clinical trial stage.

## Immunological Evaluation

Although we do not have an immunological correlate of protection with which to guide vaccine design and evaluation, there are several well-defined parameters of the host immune response which are known to be essential for protective immunity against *Mtb*. The immunological evaluation will be conducted using standardized operating procedures (SOP). Assays will include *ex vivo* IFNγ ELISpot assays, whole blood polychromatic flow cytometry with intracellular cytokine staining to measure other T cell cytokines, including IL-2, TNF-α, and IL17 from antigen-specific CD4^+^ and CD8^+^ T cells, and serological testing for antibody responses. The focus of this immunological evaluation is to use standardized assays to generate information about immune functions considered to be important in protective immunity. Work conducted within FP6 TBVAC, FP7 NEWTBVAC, and FP7 TRANSVAC has resulted in a harmonized SOP for an *ex vivo* ELISpot, which will be used here (66). In addition, a harmonized ICS flow protocol developed in these consortia will be used here as well (67). We will not use more exploratory assays in this WP, as they are less standardized, but PBMC and serum will be stored from all subjects at all time-points using harmonized SOP, so that further immunological evaluation can be performed as the field of BMs develops. In this regard, this WP will work closely with WP5 to monitor progress in the field of BM development (4, 5, 40, 41). Furthermore, ongoing work, for example, within the infrastructural FP7 EURIPRED project, to standardize the PBMC mycobacterial growth inhibition assay (MGIA), will be monitored and this assay will be conducted on cryopreserved material if the assay is deemed sufficiently fit for this purpose (68).

## Progress to Date

There are two broad categories of vaccines currently in development: “live” whole organism BCG replacement vaccines and subunit vaccines designed to boost BCG. Some live vaccines are also being evaluated as booster candidates (69, 70). It is anticipated that both types of candidate vaccines will be included and tested within this WP. Centre Hospitalier Universitaire Vaudois (CHUV), Leiden University Medical Center and University of Oxford clinical trial units will be collaborating in the planning and execution of the future candidate vaccine testing. Clinical and immunological SOPs have been agreed between the three partners. Following an open call to all partners, a first vaccine candidate has been selected and is planned to enter into phase I testing in CHUV in Q4 2017. It is expected that a second candidate will be tested in Q1 2018.

## WP5 Research into TB BMs and CoP

Next to TB vaccine development and evaluation, an important second TBVAC2020 objective is to identify TB BMs, in particular CoP. CoP are measurable BMs indicating that the host is immune, or protected against developing TB disease. Correlates of Risk (CoR) are BM indicating the host has an increased risk of developing active TB disease. Such TB BMs can be transcriptomic, cellular, or soluble analytes. The identification of CoP in particular will help to develop vaccines that target and strengthen protective immunity. Importantly, such correlates will facilitate the selection and prioritization of candidate TB vaccines for human efficacy testing, and will reduce the protracted time scale, size, and expenses of human efficacy trials by allowing the demonstration of vaccine immunogenicity and potential efficacy at an early stage. In addition, CoP will permit selection of antigens that induce protective immune responses, optimization of dose, vehicle, adjuvants, and immunization schedules of new candidate vaccines at an early stage and, thus, minimize the need for preclinical animal studies. WP5 has a strategically designed workflow, which combines TB BM discovery with parallel-specific assay development, followed by testing and validation in carefully characterized, complementary and unique human cohorts from genetically diverse populations. WP5 involves key partners from European, African, and East Asian laboratories.

**Figure 2:**
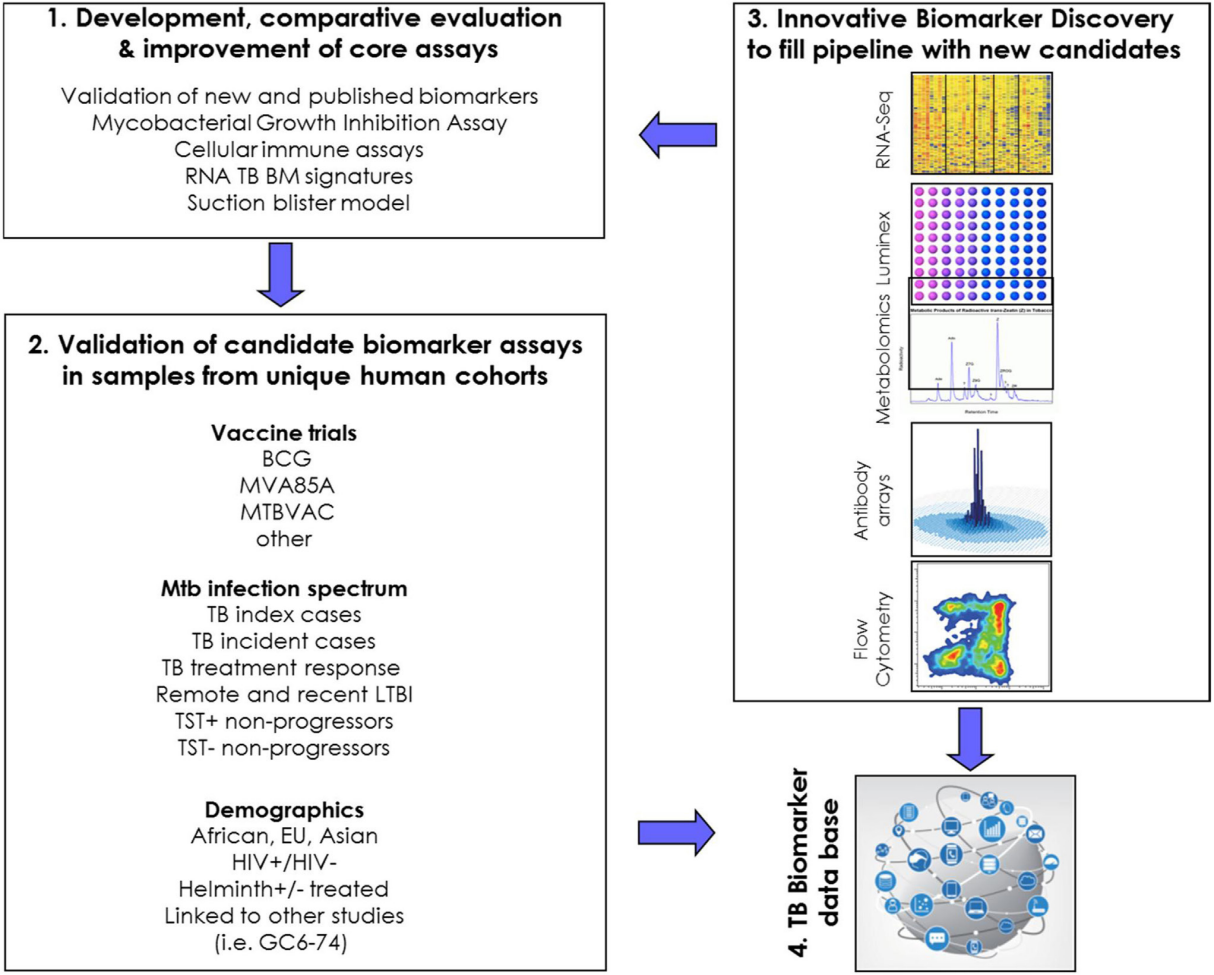
Schematic representation of research priorities and connectivity in WorkPackage 5.

This WP first aims to develop core assays for measuring CoP and CoR (#1 in Figure 2) and to evaluate these in carefully characterized, unique human cohorts from genetically diverse populations (#2 in Figure 2). Complementary to these efforts is the discovery of new TB BMs, using innovative approaches (#3 in Figure 2). A long-term goal is to move the best performing correlates forward toward validated immune correlate assays, ideally in the format of point-of-care tests for use in areas with high TB burden. Future activities will include active engagement of assay developers. Finally, the WP5 is building a database to capture the most important (transcriptomic) TB BMs (#4 in Figure 2), and there are close interactions between this WP and other efforts in this area.

**Figure 3:**
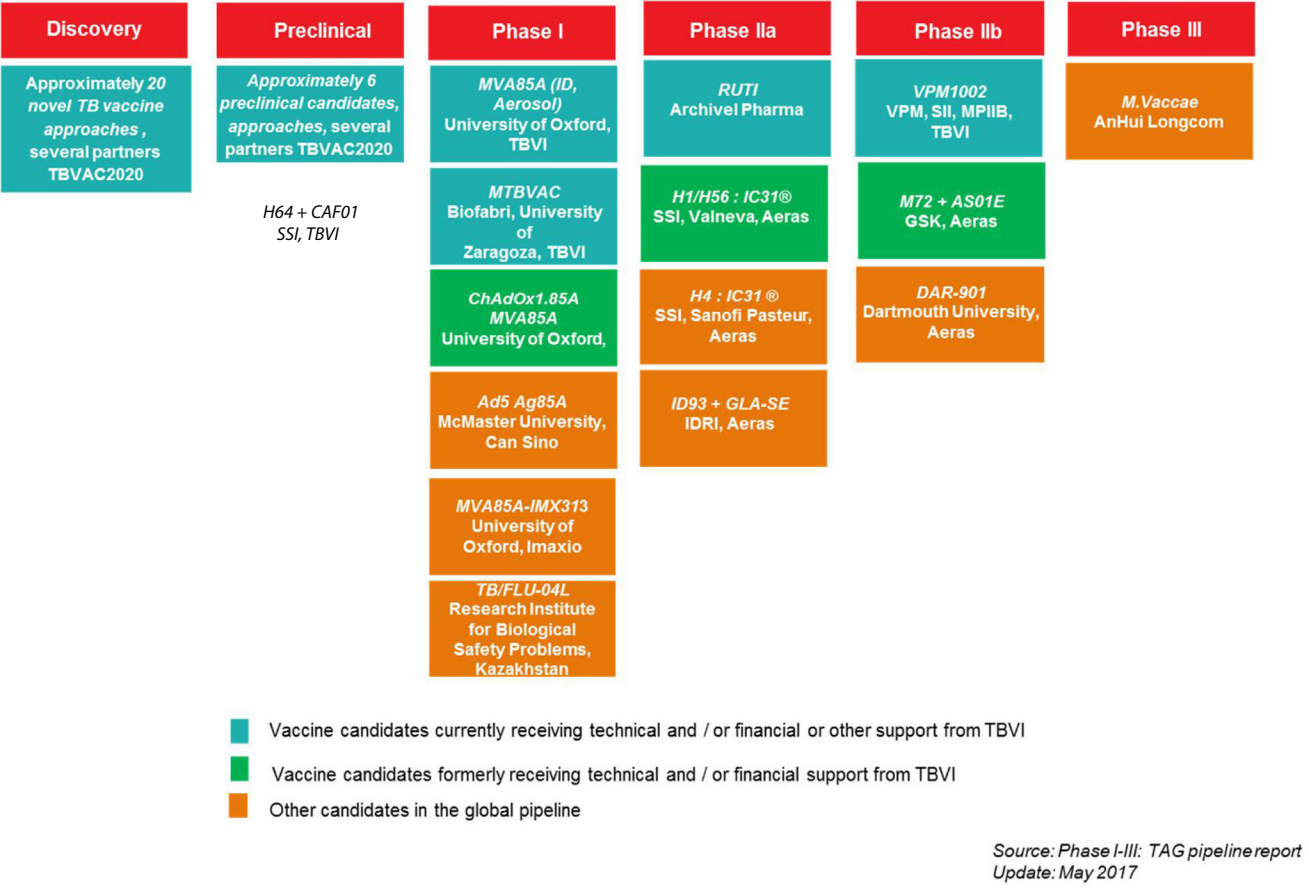
TBVI vaccine pipeline, overview of the current tuberculosis (TB) vaccine candidates in the development pipeline, per text box: acronym vaccine candidate, development partners.

**Figure 4:**
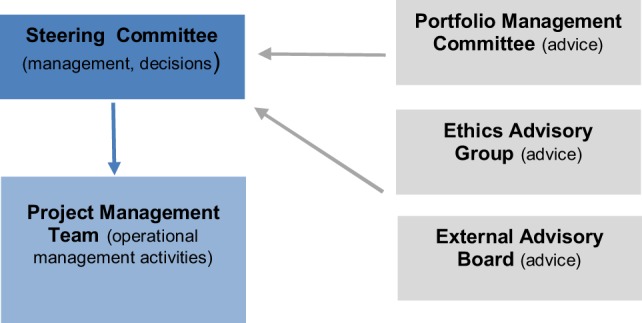
TBVAC2020 management infrastructure.

## Specific Objectives of WP5

*The first key objective is the development, evaluation, and improvement of core assays measuring CoP in TB*. To identify TB vaccine-induced CoP a protective vaccine is needed, but with the exception of BCG in settings where it does induce protection, this is not yet available. As BCG is able to induce partial protection, studies dissecting BCG-induced BMs are part of WP5. Moreover, a number of candidate correlates have recently emerged: BCG-vaccinated TB-protected South African infants had higher numbers of IFNγ-producing cells, and antibodies to *Mtb* antigen Ag85A also correlated with reduced risk of developing TB (40, 41). Other candidate CoR have been emerging (71), including myeloid/lymphoid (M/L) ratios (72), inflammation signaled by activated CD4^+^ T cells (40, 41), an IL13/AIRE ratio in HIV *Mtb* co-infected individuals (73), the lack of IFNγ responses to HBHA (74, 75), and a 16-gene based transcriptomic GC6/ACS CoR signature (76) found in large cohort studies in Africa. In other joint work by WP5 partners, diagnostic transcriptomic- or serum host protein signature-based algorithms were discovered that have powerful differential diagnostic value in diagnosing TB in both adults and children in Africa (77–79). Several research groups in WP5 are involved in efforts to further validate these correlates in larger, independent cohort studies.

In parallel to attempts identifying CoP, WP5 participants are developing unbiased, functional measures of human protective responses. One example of such an assay is the *in vitro* MGIA (68). In BCG-vaccinated infants in the UK, there was an increased ability to control the growth of BCG following BCG vaccination (43). Other “unbiased” assays include global immune-, gene expression-, and proteomic profiling-assays, which are being evaluated against outcome of infection or vaccination in clinical cohorts. We anticipate that the development and optimization of novel functional read-outs of anti-mycobacterial immunity will help identifying novel correlates, and may lead to deeper insights into potential mechanisms that control mycobacterial outgrowth. Collaborative efforts are ongoing with other EC projects, such as the FP7 EURIPRED Infrastructure project, as well as with EDCTP-funded work that focuses on TB BMs (PREDICT TB and SCREEN TB projects). Together, critical assays and assay variables are being identified to enable the development of improved, quantitative second generation assays. MGIA assays are also being studied for utility in analyzing cellular activity in human Purified Protein Derivative of *Mtb* suction blister models, in broncho-alveolar lavage (BAL) samples from aerosol BCG-infected volunteers and in the NHP model (80).

*The second objective of WP5 is the evaluation of these core assays in samples from clinical cohorts*. Such cohorts that have been collected include:

1. samples from BCG-vaccinated individuals (adults, children, neonates) from settings where BCG gives good protection against TB (northern Europe);
2. samples from trials with new candidate TB vaccines [including the MVA85 efficacy trial (81) and a recent MTBVAC trial (82)];
3. samples from TB patients with active disease (pre, during, and post treatment);
4. samples from latently TB infected individuals (LTBI), including long-term tuberculin skin test (TST) positive- as well as TST-negative- non-progressors;
5. samples from LTBI subjects co-infected with helminths pre- and post- anti-helminthic treatment;
6. samples from HIV-infected (immune-suppressed) *Mtb* co-infected subjects;
7. samples from BCG challenge models; and
8. samples and data from the unique longitudinal GC6-74 TB-progressor cohort (76).

The unique cohorts and sample banks suitable for the measurement and evaluation of correlates are available for study in WP5 and are being further expanded, including cohorts with long-term longitudinal follow-up. Multiple cohorts have been included to represent geographical, ethnic, and epidemiological diversity, with sites from South Africa, Malawi, The Gambia, Uganda, South Korea, Western/Southern Europe (UK, Netherlands, Belgium, Italy) and Eastern Europe (Belarus). This will also make it possible to test whether TB BMs, including CoR and CoP, show variation by population due to specific epidemiological settings.

*The third objective of the WP5 is the discovery of new TB candidate BM using innovative approaches*. Given the lack of CoP, BM discovery is needed to fill the TB BMs pipeline with new candidates. It is likely that multi- rather than single parameter signatures, or complex functional assays will be needed to capture the complexity and dynamics of human protective immunity to *Mtb*. Discovery approaches are focusing on both host and pathogen response during the different phases of infection. These include global transcriptomics, proteomics, metabolomics, hematopoietic (myeloid) progenitor analyses, epigenetic, and cellular approaches. In other projects partners are developing intradermal as well as aerosol BCG controlled human infection models (CHIM). Such studies will allow for identifying and measuring immune correlates, and help with correlating *in vivo* with *in vitro* results, e.g., results from CHIM with corresponding MGIA studies. To date, significant progress has been made in transcriptomic marker identification in several cohorts, with further data analysis and validation ongoing.

Assays have also been selected to survey the wider global space in the immune response to *Mtb* rather than the traditional focus on T cells and cytokines, similar to approaches in HIV correlates research. Different compartments of the “immune space” being investigated include adaptive immunity (T cells, B cells, immune regulatory cells), innate immunity [innate lymphocyte-subsets, DC-subsets, macrophages (83), tolerogenic monocytes, myeloid-derived suppressor cells (84)], NK cell subsets, dysregulation of apoptosis or autophagic flux (85), the role of particular immunometabolomic and proteomic markers (86) (in blood or other body fluids) etc. Recent findings point to an important role for: antibodies and B cells in TB (40, 41, 87, 88); a role for HLA-E restricted *Mtb* specific CD8^+^ T cells (89, 90); and polycytotoxic LAM-specific T cells (91).

The longer-term strategy of WP5 is to select the most promising markers and assays for further development toward user friendly, preferably point of care, tests (92). Partners have expertise in downstream assay development such as lateral flow assays (86) and simplified transcriptomic assays (71). Recent novel work on mathematical modeling of vaccine immune responses has shown that models can predict dose–response relationships (93).

An important point is that TB BM assays may need to be adapted to specific types of TB vaccines (e.g., live vs. subunit) or depending on the antigen and delivery system selected. This may necessitate “tailor made” sets of BMs, for which TBVI is setting up a BM Development Team (BDT).

Finally, WP5 is building a TB transcriptomic biomarker database, to be a helpful resource for the TB BM community to provide a basis for “tailor made” TB vaccine biomarker selection, and to complement a TB diagnostic BM database as a valuable resource for future research (94).

WorkPackage 5 also links to other activities in TBVAC2020, including animal models (mice, NHPs) and human clinical vaccine trial related TB BM assays; and some of WP5 partners actively participate in these activities.

## Concluding Remarks

WorkPackage 5 aims to discover new TB BM (of protection and TB risk) using innovative approaches including omics studies; to develop and improve key functional assays; and, importantly, to initially validate and then cross-validate candidate TB BMs in human cohorts (95). The long-term goal is to test and provide data on the biological validity of the BMs discovered and the assays developed using cohorts in whom protection will be demonstrable and to evaluate them against clinical endpoints (including incident TB and *Mtb* infection). The unique combination of researchers, expertise, technologies and human cohorts for validation studies is key to the success of this ambitious task.

## WP6 Portfolio Management

### Introduction

Globally, TB vaccine R&D has made tremendous progress over the last 10–15 years, from only a few TB vaccine candidates in the pipeline 15 years ago to currently more than 25 candidates in early development and 13 candidates being evaluated in clinical stages.^4^ Despite this progress, significant gaps remain that hamper efficient and effective TB vaccine development. Challenges include the establishment of:

(i) a more diverse pipeline to support successful delivery of new TB vaccines for all populations;
(ii) validated pre-clinical models predicting efficacy in humans;
(iii) validated humanCoP;
(iv) sufficient efficacy trial capacity in TB endemic areas.

TBVAC2020 directly addresses most of these challenges by supporting activities which will diversify and innovate the TB vaccine pipeline (WP1); by developing standardized animal models allowing head-to-head comparison of candidate vaccines for different disease targets (WP2); by accelerating promising preclinical vaccine candidates (WP3); by innovating clinical design and conduct phase I experimental medicine studies (WP4); and by developing CoP (WP5). The progress that TBVAC2020 is making in addressing these challenges shows that new TB vaccines are feasible. The continuation of scientific progress will require a sustained commitment over the long term for funding TB vaccine R&D by the EU and its member states. It is furthermore imperative that the TB vaccine R&D community, in turn, manages the global TB vaccine pipeline as efficiently and as coherently as possible and conducts a rationale selection of TB vaccine R&D efforts through a portfolio management approach.

### Objective

It is the objective of WP6 of the TBVAC2020 project to contribute to establishing a diverse and innovative portfolio of new TB vaccine candidates through a portfolio management approach: an objective and transparent process to prioritize and accelerate vaccine candidates in this project (Box 1).

Box 1 **Role of TBVI in accelerating the discovery and development of new tuberculosis (TB) vaccines in TBVAC2020.**

**TBVI**

TBVI is an innovation partnership that works to discover and develop new safe, effective, and affordable TB vaccines.

TBVI consist of two parts:

1. The TBVI R&D partners including more than 50 partners from academia, research institutes, and private industry in the TB vaccine field from about 20 countries in Europe, North America, Africa, Asia, and Australasia.
2. The TBVI organization that is a support structure to vaccine researchers and developers to facilitate the discovery and development of new, safe, and effective TB vaccines and biomarkers (BMs) for global use. TBVI brings together R&D organizations, scientists and industry partners in one network in order to facilitate optimal development of vaccine candidates in clinical settings. The organization provides independent technical and scientific advice to its R&D partners and funders.

TBVI provides a range of essential services to support its R&D partners as well as funders, technical agencies, and other stakeholders. These services include:

1. Technical support,
2. Knowledge development, exchange, and networking,
3. Project identification, design, development, and management,
4. Resource mobilization.

**Contribution to the global TB vaccine pipeline**

The support activities of TBVI to its TB R&D partners have been instrumental in the development of the current global TB vaccine pipeline. By facilitating discovery, preclinical, and early clinical development, TBVI has contributed to moving 50% of candidates globally to clinical stages of development (Figure 3).

1. *Technical* support *services*
  TBVI provides technical support for product and clinical development through its independent Product and Clinical Development Teams (P&CDT). This team helps manage the development of the candidates prioritized by the portfolio management process (see chapter WP6) while researchers and developers move their candidates from concept to clinical development. The P&CDT also provides advice on the experimental design of preclinical studies conducted in WorkPackage (WP) 3 and on the clinical design of the phase I trials conducted in WP4 (For more information on P&CDT see: http://www.tbvi.eu/about-us/organisation/product-and-clinical-development-team/).
2. Knowledge development, exchange, and networking.
  To optimize the discovery and development of new TB vaccines and BMs, TBVI facilitates and supports the generation of new knowledge and exchange among R&D partners by promoting knowledge-sharing through scientific meetings and workshops, publication in scientific and non-scientific journals, formal and informal networking. Through joint collaborative working with open sharing of data prior to publication, a network of trust and respect has been established between TB vaccine R&D groups throughout Europe and the world. The annual project meetings are a key example of this trust and open data sharing within the network. The annual project meeting in 2017 of the TBVAC2020 consortium organized in combination with the TBVI annual symposium brought together 163 participants from 19 countries worldwide. Beside the project R&D partners and linked industry partners, the representatives from many partner organizations, like EC, Aeras, European & Developing Countries Clinical Trials Partnership, Bill & Melinda Gates Foundation (BMGF), World Health Organization, European Research Infrastructure for Translational Medicine and European Vaccine Initiative attended this meeting.
3. Project identification, design, development, and management.
  TBVI initiates new projects when new funding opportunities arise. In line with its R&D strategy, TBVI applies a bottom-up process working closely with its partners to develop project proposals. An example of this process was the creation of the TBVAC2020 project proposal. TBVI identified the European Commission (EC) Horizon 2020 call on new TB vaccines. In consultation with leading experts in the field, TBVI identified the areas and scope of work to be included in the project proposal. To enable the involvement of new partners and to receive the best new ideas, TBVI launched an open call for Expression of Interest (EoI). Over 100 EoI letters were received. A project selection committee selected the best project proposals based on predetermined selection criteria. This finally resulted in EC awarding €18.2 million for the TBVAC2020 project. Additional funds from the Swiss, Korean, and Australian governments complemented the project budget to a total of €24.6 million (http://horizon2020projects.com/sc-health/tb-consortium-awarded-e24m-international-grant/).
  TBVI is the coordinator of the TBVAC2020 project and provides essential services to contribute to successful completion of the pre-set project deliverables and milestones, to increase the overall impact of the project, and to ensure coordination and linkages between TBVAC2020 and other TB vaccine R&D initiatives. To ensure efficient and effective management of the project, TBVI has established and implemented the management infrastructure (Figure 4). The project infrastructure is composed of the Steering Committee, the Project Management Team (PMT led by the Coordinator) and the following three advisory groups: Portfolio Management Committee, Ethics advisory group, and External Advisory Committee.
4. Resource mobilization.
  TBVI mobilizes resources for its R&D partners. TBVI was able to leverage funding from this project with other funders including Norwegian Agency for Development Cooperation, Department of International Development and BMGF. This enabled additional and accelerated development of two preclinical and three clinical stage candidates, of two novel and refined preclinical models, and made it possible to continue key research and development activities on correlates of protection of 7 partners.

### Portfolio Management

Portfolio management is an efficient and effective mechanism to advance the vaccine pipeline. It is a quality decision-making process seeking to maximize probability of success against acceptable cost and risk. Portfolio management ensures alignment of vaccine candidates and resources, and ensures an optimal balance between them (96). The aim of WP6 is to contribute to the establishment of a balanced and diverse portfolio of preclinical and early clinical TB vaccine candidates with prospective applications in infants, adolescents, and adults that focus on either preventing active TB disease or improving treatment of TB disease.

The portfolio management approach applied in TBVAC2020 is based on three sets of criteria:

1. Stage-gating criteria. Stage-gating criteria provide a linear assessment of each individual candidate to determine if there is sufficient robust evidence to support advancement to the next stage of development (34, 97).
2. Portfolio assessment. Priority-setting assessment criteria allow comparison of similar vaccine candidates in a portfolio to select and deselect candidate(s), especially those at the same developmental stage.
3. Entry of new candidates. Entry criteria allow assessment of new candidates into the portfolio to create more diversity and balance in the portfolio and to increase chance of success.

An independent PMC uses these three sets of criteria to monitor and provide advice to the Steering Committee of TBVAC2020 on the vaccine pipeline of the project. It monitors discovery activities in WP1 and WP2, provides advice on entrance of new candidates, advises on selection and priority of vaccine candidates for evaluation in preclinical models or to support their preclinical development (WP3), and for selection and evaluation of candidates in clinical trials (WP4). During the first 30 months of the TBVAC2020 project, PMC advised on the selection of 21 candidate vaccines for evaluation in 6 head-to-head preclinical models, 4 candidates for receiving support for preclinical development among others formulation, Good Manufacturing Practice (GMP) and developing a product development plan, and two candidates for evaluation in a phase I clinical trial. During this period, PMC deselected six vaccine candidates for evaluation in the head-to-head preclinical models and 1 candidate was deselected for evaluation in a phase I clinical trial. It is the intention that PMC of WP6 will integrate with the Global Portfolio Review Committee (GPRC) for the Global TB vaccine Partnership (GTBVP) once this entity is established. The GPRC aims to provide advice from a global perspective, with regard to selection or deselection of vaccine candidates for support to the next step in development.

## Contributors: Participating Investigators in TBVAC2020 Consortium

The TBVAC2020 consortium consists of 42 partner institutions from 15 countries, including 10 from Europe, 1 from USA, 1 from South Korea, 1 from Australia, 1 from the Gambia, and 1 from South Africa represented by the following members:

**Australia:** Warwick Britton (W.J. Britton), University of Sydney, Autralia; Jamie Triccas (J.A. Triccas), University of Sydney, Autralia; Claudio Counoupas (C. Counoupas), University of Sydney, Autralia. **Belgium:** Johan Grooten (J. Grooten), Ghent University, Belgium; Marie-Ange Demoitie (M.A. Demoitié), GSK Biologicals, Belgium; Marta Romano (M. Romano), Scientific Institute of Public Health (WIV-ISP), Belgium; Kris Huygen (K. Huygen), Scientific Institute of Public Health (WIV-ISP), Belgium; Hermann Giresse Tima (HG. Tima), Scientific Institute of Public Health (WIV-ISP), Belgium; Francoise Mascart (F. Mascart) , Université Libre de Bruxelles, Belgium. **Denmark:** Peter Andersen (P.L. Andersen), Statens Serum Institut (SSI); Claus Aagaard (C. Aagaard), Statens Serum Institut (SSI); Dennis Christensen (D. Christensen), Statens Serum Institut (SSI); Morten Ruhwald (M. Ruhwald), Statens Serum Institut (SSI); Thomas Lindenstrom (T. Lindenstrom), Statens Serum Institut (SSI). **France:** Olivier Neyrolles (O. Neyrolles), Centre National de la Recherche Scientifique (CNRS); Pierre Charneau (P. Charneau), Institut Pasteur Paris; Christophe Guilhot (C. Guilhot), Centre National de la Recherche Scientifique (CNRS); Antonio Peixoto (A. Peixoto), Centre National de la Recherche Scientifique (CNRS); Martine Gilleron (M. Gilleron), Centre National de la Recherche Scientifique (CNRS); Isabelle Vergne (I. Vergne), Centre National de la Recherche Scientifique (CNRS); Camille Locht (C. Locht), Institut Pasteur de Lille; Roland Brosch (R. Brosch), Institut Pasteur Paris; Genevieve Inchauspe (G. Inchauspé), Transgene; Stephane Leung Theung Long (S. Leung-Theung-Long), Transgene. **Germany:** Stefan Kaufmann (S.H.E. Kaufmann), Max Planck Institute for Infection Biology; January Weiner (J. Weiner), Max Planck Institute for Infection Biology; Jeroen Maertzdorf (J. Maertzdorf), Max Planck Institute for Infection Biology; Natalie Nieuwenhuizen (N. Nieuwenhuizen), Max Planck Institute for Infection Biology; Max Bastian (M. Bastian), Friedrich-Loeffler-Institut; Steffen Stenger (S. Stenger), University of Ulm; Stephanie Kallert (S. Kallert), University of Ulm. **Ireland:** Stephen Gordon (S.V. Gordon), University College Dublin. **Italy:** Nadia Caccamo (N. Caccomo), Azienda Ospedaliera Universitaria Policlinico “Paolo Giaccone” di Palermo; Delia Goletti (D. Goletti), IRCCS Lazzaro Spallanzani; Roberto Nisini (R. Nisini), Istituto Superiore Di Sanità (ISS). **Republic of Korea:** Sung Jae Shin (S.J. Shin), Yonsei University College of Medicine; Sang Nae Cho (Ray) (S.N. Cho), Yonsei University College of Medicine; Hyejon Lee (H. Lee), International Tuberculosis Research Center; Ino Choi (I. Choi), International Tuberculosis Research Center. **South Africa:** Alex Sigal (A. Sigal), Kwazulu-Natal Research Institute for Tuberculosis; Thomas Scriba (T.J. Scriba), South African Tuberculosis Vaccine Initiative; Gerhard Walzl (G. Walzl), Stellenbosch University; Andre Loxton (A.G. Loxton), Stellenbosch University; Robert Wilkinson (R.J. Wilkinson), University of Cape Town; Katalin Wilkinson (K.A. Wilkinsons), University of Cape Town. **Spain:** Pere-Joan Cardona (P.J. Cardona), Fundació Institut d’ Investigació en Ciències de la Salut Germans Trias i Pujol; Cris Vilaplana (C. Vilaplana), Fundació Institut d’ Investigació en Ciències de la Salut Germans Trias i Pujol; Carlos Martin (C. Martin), University of Zaragoza; Dessi Marinova (D.V. Marinova), University of Zaragoza; Nacho Aguilo (N. Aguilo), University of Zaragoza. **Switzerland:** François Spertini (F. Spertini), Centre Hospitalier Universitaire Vaudois; Ruedi Aebersold (R. Aebersold), ETH Zürich – Institute of Molecular Systems Biology; Etienne Caron (E. Caron), ETH Zürich – Institute of Molecular Systems Biology; Daniel Pinschewer (D. Pinschewer), University of Basel; Gennaro De Libero (G. De Libero), University of Basel; Claire Anne Siegrist (C.A. Siegrist), University of Geneva; Nicolas Collin (N. Collin), University of Lausanne; Christophe Barnier-Quer (C. Barnier-Quer), University of Lausanne; Peter Sander (P. Sander), University of Zürich. **The Gambia:** Jayne Sutherland (J.S. Sutherland), Medical Research Council. **The Netherlands:** Frank Verreck (F.A.W. Ferreck), Biomedical Primate Research Centre (BPRC); Tom Ottenhoff (T.H.M. Ottenhoff), Leiden University Medical Center (LUMC); Simone Joosten (S.A. Joosten), Leiden University Medical Center (LUMC); Krista van Meijgaarden, Leiden University Medical Center (LUMC); Mariateresa Coppola, Leiden University Medical Center (LUMC); Annemieke Geluk, Leiden University Medical Center (LUMC); Nick Drager (N. Drager), Tuberculosis Vaccine Initiative (TBVI); Danielle Roordink (D.M. Roordink), Tuberculosis Vaccine Initiative (TBVI); Jelle Thole (J. Thole), Tuberculosis Vaccine Initiative (TBVI). **United Kingdom:** Yvonne Perrie (Y. Perrie), University of Strathclyde; Marc Baird (M.S. Baird), Bangor University; Michael Levin (M. Levin), Imperial College, Faculty of Medicine, Department of Medicine & School of Public Health; Myrsini Kaforou (M. Kaforou), Imperial College, Faculty of Medicine, Department of Medicine & School of Public Health; Hazel Dockrell (H.M. Dockrell), London School of Hygiene and Tropical Medicine (LSHTM); Steven Smith (S.G. Smith), London School of Hygiene and Tropical Medicine (LSHTM); Helen Fletcher (H.A. Fletcher), London School of Hygiene and Tropical Medicine (LSHTM); Gregory Bancroft (G.J. Bancroft), London School of Hygiene and Tropical Medicine (LSHTM); Ann Rawkins (A. Williams), Public Health England; Simon Clark (S. Clark), Public Health England; Sally Sharpe (S. Sharpe), Public Health England; Mei Mei Ho (M.M. Ho), The Department of Health, UK, acting through PHE and MHRA; Helen McShane (H. McShane), University of Oxford; Iman Satti (I. Satti), University of Oxford; Elena Stylianou (E. Stylianou), University of Oxford; Rachel Tanner (R. Tanner), University of Oxford; Martin Vordermeier (H.M. Vordermeier), Animal and Plant Health Agency (APHA); Philip Hogarth (P.J. Hogarth), Animal and Plant Health Agency (APHA). **United States of America:** Danilo Casimiro (D. Casimiro), Aeras.

The mentioned investigators have all conducted and/or contributed to the R&D activities of TBVAC2020 project described in this manuscript.

## Author Contributions

All authors contributed to the writing and editing of the manuscript.

## Conflict of Interest Statement

SK is co-inventor of BCG DureC::hly (VPM1002). BP is affiliated with RegExcel Consulting Ltd. All other authors have no other potential conflicts of interest to disclose.

## Footnotes

^1^www.aeras.org.

^2^www.emi-tb.org.

^3^https://www.ctvd.co.

^4^http://www.tbvi.eu/what-we-do/pipeline-of-vaccines/.
